# Supplementary material for: Role and mechanism of NCAPD3 in promoting malignant behaviors in gastric cancer
Source: Front Pharmacol. 2024 Apr 22;15:1341039. doi: 10.3389/fphar.2024.1341039 (PMC11070777; doi:10.3389/fphar.2024.1341039)
Supplement: Supplementary file 11 [file DataSheet2.ZIP › GSEA/Transcipt factor/my_analysis.Gsea.1599461814734/ZNF407_TARGET_GENES.html]

Details for gene set ZNF407\_TARGET\_GENES[GSEA]

|  || Dataset | filtered\_dataset.sample\_info.cls#WT\_versus\_NCAPD3\_MUT |
| Phenotype | sample\_info.cls#WT\_versus\_NCAPD3\_MUT |
| Upregulated in class | WT |
| GeneSet | ZNF407\_TARGET\_GENES |
| Enrichment Score (ES) | 0.21645738 |
| Normalized Enrichment Score (NES) | 1.6098183 |
| Nominal p-value | 0.004285714 |
| FDR q-value | 0.5624504 |
| FWER p-Value | 1.0 |
Table: GSEA Results Summary

  

Fig 1: Enrichment plot: ZNF407\_TARGET\_GENES      
 Profile of the Running ES Score & Positions of GeneSet Members on the Rank Ordered List

  

| SYMBOL | TITLE | RANK IN GENE LIST | RANK METRIC SCORE | RUNNING ES | CORE ENRICHMENT || 1 | 2935 | GSPT1 | 5 | 1.156 | 0.0097 | Yes |
| 2 | 5514 | PPP1R10 | 11 | 1.087 | 0.0187 | Yes |
| 3 | 55149 | MTPAP | 15 | 1.051 | 0.0288 | Yes |
| 4 | 23248 | RPRD2 | 33 | 0.977 | 0.0268 | Yes |
| 5 | 80746 | TSEN2 | 53 | 0.909 | 0.0224 | Yes |
| 6 | 23322 | RPGRIP1L | 60 | 0.871 | 0.0280 | Yes |
| 7 | 285989 | ZNF789 | 66 | 0.857 | 0.0342 | Yes |
| 8 | 55794 | DDX28 | 68 | 0.856 | 0.0436 | Yes |
| 9 | 153364 | MBLAC2 | 79 | 0.838 | 0.0455 | Yes |
| 10 | 81839 | VANGL1 | 98 | 0.790 | 0.0406 | Yes |
| 11 | 7769 | ZNF226 | 125 | 0.777 | 0.0291 | Yes |
| 12 | 390927 | ZNF793 | 137 | 0.758 | 0.0293 | Yes |
| 13 | 55900 | ZNF302 | 145 | 0.740 | 0.0325 | Yes |
| 14 | 9848 | MFAP3L | 155 | 0.726 | 0.0340 | Yes |
| 15 | 162967 | ZNF320 | 156 | 0.726 | 0.0426 | Yes |
| 16 | 7248 | TSC1 | 158 | 0.718 | 0.0503 | Yes |
| 17 | 54971 | BANP | 172 | 0.707 | 0.0483 | Yes |
| 18 | 57325 | CSRP2BP | 176 | 0.698 | 0.0542 | Yes |
| 19 | 613 | BCR | 180 | 0.695 | 0.0601 | Yes |
| 20 | 9897 | KIAA0196 | 197 | 0.682 | 0.0554 | Yes |
| 21 | 55798 | METTL2B | 199 | 0.678 | 0.0627 | Yes |
| 22 | 145389 | SLC38A6 | 202 | 0.673 | 0.0691 | Yes |
| 23 | 22948 | CCT5 | 215 | 0.663 | 0.0674 | Yes |
| 24 | 127253 | TYW3 | 225 | 0.654 | 0.0680 | Yes |
| 25 | 400410 | ST20 | 235 | 0.647 | 0.0685 | Yes |
| 26 | 8831 | SYNGAP1 | 236 | 0.647 | 0.0762 | Yes |
| 27 | 221504 | ZBTB9 | 237 | 0.647 | 0.0839 | Yes |
| 28 | 57521 | RPTOR | 239 | 0.645 | 0.0907 | Yes |
| 29 | 6426 | SRSF1 | 243 | 0.637 | 0.0959 | Yes |
| 30 | 55745 | AP5M1 | 246 | 0.634 | 0.1018 | Yes |
| 31 | 528 | ATP6V1C1 | 254 | 0.625 | 0.1037 | Yes |
| 32 | 87178 | PNPT1 | 263 | 0.622 | 0.1047 | Yes |
| 33 | 116143 | WDR92 | 267 | 0.619 | 0.1096 | Yes |
| 34 | 22845 | DOLK | 269 | 0.618 | 0.1162 | Yes |
| 35 | 100302736 | TMED7-TICAM2 | 270 | 0.617 | 0.1235 | Yes |
| 36 | 199857 | ALG14 | 275 | 0.615 | 0.1276 | Yes |
| 37 | 23341 | DNAJC16 | 276 | 0.613 | 0.1349 | Yes |
| 38 | 84874 | ZNF514 | 283 | 0.606 | 0.1373 | Yes |
| 39 | 23036 | ZNF292 | 288 | 0.602 | 0.1413 | Yes |
| 40 | 25972 | UNC50 | 297 | 0.593 | 0.1419 | Yes |
| 41 | 81889 | FAHD1 | 299 | 0.592 | 0.1482 | Yes |
| 42 | 36 | ACADSB | 302 | 0.589 | 0.1536 | Yes |
| 43 | 7626 | ZNF75D | 309 | 0.588 | 0.1558 | Yes |
| 44 | 7014 | TERF2 | 323 | 0.579 | 0.1523 | Yes |
| 45 | 55697 | VAC14 | 329 | 0.576 | 0.1551 | Yes |
| 46 | 3149 | HMGB3 | 331 | 0.575 | 0.1612 | Yes |
| 47 | 4297 | KMT2A | 337 | 0.573 | 0.1640 | Yes |
| 48 | 100529261 | CHURC1-FNTB | 343 | 0.570 | 0.1668 | Yes |
| 49 | 2342 | FNTB | 344 | 0.570 | 0.1735 | Yes |
| 50 | 55676 | SLC30A6 | 353 | 0.565 | 0.1739 | Yes |
| 51 | 2965 | GTF2H1 | 356 | 0.563 | 0.1790 | Yes |
| 52 | 5810 | RAD1 | 360 | 0.560 | 0.1832 | Yes |
| 53 | 23184 | MESDC2 | 362 | 0.559 | 0.1891 | Yes |
| 54 | 5036 | PA2G4 | 365 | 0.557 | 0.1941 | Yes |
| 55 | 166785 | MMAA | 369 | 0.553 | 0.1982 | Yes |
| 56 | 326625 | MMAB | 371 | 0.552 | 0.2040 | Yes |
| 57 | 10618 | TGOLN2 | 383 | 0.547 | 0.2017 | Yes |
| 58 | 9815 | GIT2 | 394 | 0.540 | 0.2002 | Yes |
| 59 | 54994 | GID8 | 415 | 0.526 | 0.1905 | Yes |
| 60 | 84274 | COQ5 | 419 | 0.523 | 0.1943 | Yes |
| 61 | 54665 | RSBN1 | 429 | 0.516 | 0.1932 | Yes |
| 62 | 10885 | WDR3 | 430 | 0.515 | 0.1993 | Yes |
| 63 | 54482 | TRMT13 | 449 | 0.506 | 0.1910 | Yes |
| 64 | 23192 | ATG4B | 452 | 0.505 | 0.1954 | Yes |
| 65 | 2920 | CXCL2 | 454 | 0.501 | 0.2006 | Yes |
| 66 | 146691 | TOM1L2 | 472 | 0.490 | 0.1928 | Yes |
| 67 | 55705 | IPO9 | 476 | 0.487 | 0.1962 | Yes |
| 68 | 23116 | FAM179B | 482 | 0.486 | 0.1980 | Yes |
| 69 | 140775 | SMCR8 | 487 | 0.480 | 0.2005 | Yes |
| 70 | 5510 | PPP1R7 | 490 | 0.478 | 0.2046 | Yes |
| 71 | 3184 | HNRNPD | 508 | 0.470 | 0.1966 | Yes |
| 72 | 54920 | DUS2 | 509 | 0.470 | 0.2022 | Yes |
| 73 | 130502 | TTC32 | 517 | 0.465 | 0.2022 | Yes |
| 74 | 204 | AK2 | 529 | 0.458 | 0.1988 | Yes |
| 75 | 84081 | NSRP1 | 538 | 0.453 | 0.1978 | Yes |
| 76 | 8195 | MKKS | 542 | 0.449 | 0.2008 | Yes |
| 77 | 3609 | ILF3 | 544 | 0.449 | 0.2053 | Yes |
| 78 | 7334 | UBE2N | 546 | 0.449 | 0.2098 | Yes |
| 79 | 9836 | LCMT2 | 549 | 0.448 | 0.2136 | Yes |
| 80 | 9416 | DDX23 | 553 | 0.445 | 0.2165 | Yes |
| 81 | 55317 | AP5S1 | 573 | 0.437 | 0.2065 | No |
| 82 | 57579 | FAM135A | 577 | 0.436 | 0.2093 | No |
| 83 | 23786 | BCL2L13 | 588 | 0.432 | 0.2064 | No |
| 84 | 26057 | ANKRD17 | 589 | 0.431 | 0.2115 | No |
| 85 | 10445 | MCRS1 | 617 | 0.414 | 0.1949 | No |
| 86 | 11083 | DIDO1 | 631 | 0.406 | 0.1894 | No |
| 87 | 57679 | ALS2 | 656 | 0.399 | 0.1750 | No |
| 88 | 375248 | ANKRD36 | 674 | 0.389 | 0.1660 | No |
| 89 | 55157 | DARS2 | 686 | 0.385 | 0.1618 | No |
| 90 | 285636 | C5orf51 | 692 | 0.382 | 0.1624 | No |
| 91 | 10452 | TOMM40 | 696 | 0.380 | 0.1645 | No |
| 92 | 6651 | SON | 716 | 0.365 | 0.1537 | No |
| 93 | 23350 | U2SURP | 719 | 0.364 | 0.1564 | No |
| 94 | 55759 | WDR12 | 731 | 0.359 | 0.1519 | No |
| 95 | 134430 | WDR36 | 733 | 0.359 | 0.1554 | No |
| 96 | 128061 | C1orf131 | 738 | 0.356 | 0.1564 | No |
| 97 | 81627 | TRMT1L | 745 | 0.353 | 0.1558 | No |
| 98 | 90120 | C9orf69 | 758 | 0.343 | 0.1504 | No |
| 99 | 3188 | HNRNPH2 | 769 | 0.338 | 0.1464 | No |
| 100 | 54939 | COMMD4 | 777 | 0.335 | 0.1448 | No |
| 101 | 10492 | SYNCRIP | 779 | 0.334 | 0.1480 | No |
| 102 | 51575 | ESF1 | 812 | 0.309 | 0.1261 | No |
| 103 | 9343 | EFTUD2 | 818 | 0.305 | 0.1257 | No |
| 104 | 11091 | WDR5 | 822 | 0.304 | 0.1270 | No |
| 105 | 26121 | PRPF31 | 825 | 0.301 | 0.1289 | No |
| 106 | 387680 | FAM21A | 828 | 0.297 | 0.1309 | No |
| 107 | 253725 | FAM21C | 829 | 0.297 | 0.1344 | No |
| 108 | 11157 | LSM6 | 830 | 0.296 | 0.1379 | No |
| 109 | 3673 | ITGA2 | 858 | -0.264 | 0.1195 | No |
| 110 | 5723 | PSPH | 859 | -0.267 | 0.1227 | No |
| 111 | 7296 | TXNRD1 | 877 | -0.289 | 0.1126 | No |
| 112 | 990 | CDC6 | 888 | -0.304 | 0.1082 | No |
| 113 | 27230 | SERP1 | 892 | -0.306 | 0.1095 | No |
| 114 | 64063 | PRSS22 | 896 | -0.315 | 0.1108 | No |
| 115 | 51141 | INSIG2 | 903 | -0.328 | 0.1099 | No |
| 116 | 27131 | SNX5 | 907 | -0.333 | 0.1115 | No |
| 117 | 51124 | IER3IP1 | 918 | -0.347 | 0.1076 | No |
| 118 | 6273 | S100A2 | 942 | -0.365 | 0.0936 | No |
| 119 | 9572 | NR1D1 | 977 | -0.392 | 0.0712 | No |
| 120 | 84919 | PPP1R15B | 979 | -0.394 | 0.0751 | No |
| 121 | 10724 | MGEA5 | 983 | -0.394 | 0.0774 | No |
| 122 | 7090 | TLE3 | 984 | -0.395 | 0.0820 | No |
| 123 | 10938 | EHD1 | 1019 | -0.419 | 0.0599 | No |
| 124 | 57552 | NCEH1 | 1020 | -0.419 | 0.0649 | No |
| 125 | 3516 | RBPJ | 1021 | -0.419 | 0.0698 | No |
| 126 | 7871 | SLMAP | 1038 | -0.435 | 0.0623 | No |
| 127 | 130617 | ZFAND2B | 1046 | -0.444 | 0.0619 | No |
| 128 | 80315 | CPEB4 | 1053 | -0.448 | 0.0625 | No |
| 129 | 7132 | TNFRSF1A | 1055 | -0.449 | 0.0670 | No |
| 130 | 5889 | RAD51C | 1059 | -0.451 | 0.0700 | No |
| 131 | 81631 | MAP1LC3B | 1060 | -0.452 | 0.0754 | No |
| 132 | 2523 | FUT1 | 1068 | -0.456 | 0.0752 | No |
| 133 | 283 | ANG | 1074 | -0.458 | 0.0766 | No |
| 134 | 1657 | DMXL1 | 1080 | -0.462 | 0.0781 | No |
| 135 | 604 | BCL6 | 1136 | -0.504 | 0.0403 | No |
| 136 | 51105 | PHF20L1 | 1140 | -0.506 | 0.0439 | No |
| 137 | 11132 | CAPN10 | 1141 | -0.508 | 0.0499 | No |
| 138 | 221491 | C6orf1 | 1144 | -0.508 | 0.0544 | No |
| 139 | 84925 | DIRC2 | 1153 | -0.516 | 0.0541 | No |
| 140 | 92014 | SLC25A51 | 1155 | -0.519 | 0.0595 | No |
| 141 | 9411 | ARHGAP29 | 1174 | -0.539 | 0.0515 | No |
| 142 | 54407 | SLC38A2 | 1182 | -0.553 | 0.0525 | No |
| 143 | 6038 | RNASE4 | 1194 | -0.570 | 0.0505 | No |
| 144 | 7128 | TNFAIP3 | 1198 | -0.573 | 0.0549 | No |
| 145 | 5504 | PPP1R2 | 1199 | -0.576 | 0.0618 | No |
| 146 | 57649 | PHF12 | 1212 | -0.588 | 0.0592 | No |
| 147 | 29943 | PADI1 | 1225 | -0.600 | 0.0567 | No |
| 148 | 2005 | ELK4 | 1235 | -0.612 | 0.0568 | No |
| 149 | 22809 | ATF5 | 1246 | -0.624 | 0.0563 | No |
| 150 | 8412 | BCAR3 | 1261 | -0.648 | 0.0528 | No |
| 151 | 9262 | STK17B | 1268 | -0.659 | 0.0558 | No |
| 152 | 83931 | STK40 | 1281 | -0.669 | 0.0542 | No |
| 153 | 1719 | DHFR | 1298 | -0.700 | 0.0498 | No |
| 154 | 8744 | TNFSF9 | 1305 | -0.708 | 0.0534 | No |
| 155 | 23365 | ARHGEF12 | 1329 | -0.751 | 0.0440 | No |
| 156 | 55521 | TRIM36 | 1337 | -0.774 | 0.0476 | No |
| 157 | 23431 | AP4E1 | 1359 | -0.827 | 0.0407 | No |
Table: GSEA details [plain text format]

  

Fig 2: ZNF407\_TARGET\_GENES      
 Blue-Pink O' Gram in the Space of the Analyzed GeneSet

  

Fig 3: ZNF407\_TARGET\_GENES: Random ES distribution      
 Gene set null distribution of ES for **ZNF407\_TARGET\_GENES**
